# Epigenome-wide association study of whole blood gene expression in Framingham Heart Study participants provides molecular insight into the potential role of CHRNA5 in cigarette smoking-related lung diseases

**DOI:** 10.1186/s13148-021-01041-5

**Published:** 2021-03-22

**Authors:** Chen Yao, Roby Joehanes, Rory Wilson, Toshiko Tanaka, Luigi Ferrucci, Anja Kretschmer, Holger Prokisch, Katharina Schramm, Christian Gieger, Annette Peters, Melanie Waldenberger, Carola Marzi, Christian Herder, Daniel Levy

**Affiliations:** 1 The Framingham Heart Study, 73 Mt. Wayte Avenue, Framingham, MA 01702 USA; 2 The Population Sciences Branch, Division of Intramural Research, National Heart, Lung, and Blood Institute, Bethesda, MD USA; 3 Research Unit Molecular Epidemiology, Helmholtz Zentrum München, German Research Center for Environmental Health, 85764 Bavaria, Neuherberg Germany; 4 Institute of Epidemiology, Helmholtz Zentrum München, German Research Center for Environmental Health, 85764 Bavaria, Neuherberg Germany; 5 Longitudinal Study Section, National Institute On Aging, Baltimore, MD USA; 6 Institute of Human Genetics, Helmholtz Center Munich, German Research Center for Environmental Health, Neuherberg, Germany; 7 Institute of Human Genetics, Technical University Munich, München, Germany; 8 Institute for Neurogenomics, Helmholtz Center Munich, German Research Center for Environmental Health, Neuherberg, Germany; 9 Institute of Genetic Epidemiology, Helmholtz Zentrum München - German Research Center for Environmental Health, 85764 Neuherberg, Germany; 10 Chair of Genetic Epidemiology, IBE, Faculty of Medicine, LMU Munich, 81377 Munich, Germany; 11 Department of Internal Medicine I (Cardiology), Hospital of the Ludwig-Maximilians-University (LMU) Munich, 81377 Munich, Germany; 12 German Center for Cardiovascular Research (DZHK), Partner Site Munich Heart Alliance, Munich, Germany; 13 Research Unit of Molecular Epidemiology, Institute of Epidemiology II, German Research Center for Environmental Health, Neuherberg, Germany; 14 Institute for Clinical Diabetology, German Diabetes Center, Leibniz Center for Diabetes Research At Heinrich Heine University Düsseldorf, Düsseldorf, Germany; 15 German Center for Diabetes Research (DZD), Partner Düsseldorf, Germany; 16 Division of Endocrinology and Diabetology, Medical Faculty, Heinrich Heine University, Düsseldorf, Germany

**Keywords:** EWAS, CHRNA5, Smoking, Lung cancer, COPD, Mendelian randomization

## Abstract

**Background:**

DNA methylation is a key epigenetic modification that can directly affect gene regulation. DNA methylation is highly influenced by environmental factors such as cigarette smoking, which is causally related to chronic obstructive pulmonary disease (COPD) and lung cancer. To date, there have been few large-scale, combined analyses of DNA methylation and gene expression and their interrelations with lung diseases.

**Results:**

We performed an epigenome-wide association study of whole blood gene expression in ~ 6000 individuals from four cohorts. We discovered and replicated numerous CpGs associated with the expression of *cis* genes within 500 kb of each CpG, with 148 to 1,741 *cis* CpG-transcript pairs identified across cohorts. We found that the closer a CpG resided to a transcription start site, the larger its effect size, and that 36% of *cis* CpG-transcript pairs share the same causal genetic variant. Mendelian randomization analyses revealed that hypomethylation and lower expression of *CHRNA5*, which encodes a smoking-related nicotinic receptor, are causally linked to increased risk of COPD and lung cancer. This putatively causal relationship was further validated in lung tissue data.

**Conclusions:**

Our results provide a large and comprehensive association study of whole blood DNA methylation with gene expression. Expression platform differences rather than population differences are critical to the replication of *cis* CpG-transcript pairs. The low reproducibility of *trans* CpG-transcript pairs suggests that DNA methylation regulates nearby rather than remote gene expression. The putatively causal roles of methylation and expression of *CHRNA5* in relation to COPD and lung cancer provide evidence for a mechanistic link between patterns of smoking-related epigenetic variation and lung diseases, and highlight potential therapeutic targets for lung diseases and smoking cessation.

**Supplementary Information:**

The online version contains supplementary material available at 10.1186/s13148-021-01041-5.

## Background

The effects of environmental exposures on downstream phenotypes are mediated in part by DNA methylation [1]. DNA methylation was long thought to inhibit gene expression [2]. Recent studies, however, have revealed a more complex picture. DNA methylation levels have been shown to be inversely correlated with gene expression across the genome and throughout multiple cell types, but site-specific analyses have revealed positive correlations of DNA methylation with the expression of some genes [3]. CpG sites, locations in the genome where a cytosine is followed by a guanine nucleotide, are often methylated, and CpG sites whose methylation is associated with altered gene expression are referred to as expression quantitative trait methylation sites (eQTMs). CpG sites that are positively correlated with gene expression may act by different mechanisms compared with CpGs that are inversely correlated with expression. As a general feature across different cell types, CpG sites that are inversely correlated with gene expression are significantly more likely to be found in transcriptional repressor CCCTC-binding factor (CTCF) binding sites, enhancers, and promoters, particularly non-CpG island (CGI) promoters, whereas positively correlated CpG sites are more likely to be found in gene bodies [4].

DNA methylation has been studied genome-wide in relation to a wide range of phenotypes, with numerous associations having been reported, including with cancer, autoimmune disease, diabetes, cardiovascular disease, and neurological diseases [5–10]. Association, however, does not prove causation. Most disease-associated methylation changes have been found to be a consequence of the traits studied and are not causal of disease [11, 12]. Moreover, DNA methylation is determined by a complex interplay of genetic and environmental factors. In particular, current and prior cigarette smoking have a profound influence on the methylation levels of thousands of CpGs [13]. Mendelian randomization (MR) has been proposed as a means to infer causal relations between DNA methylation and disease outcomes [14]. This approach uses a genetic proxy as an instrument to represent DNA methylation and to evaluate the likelihood of a causal association between DNA methylation and disease.

**Fig. 1:**
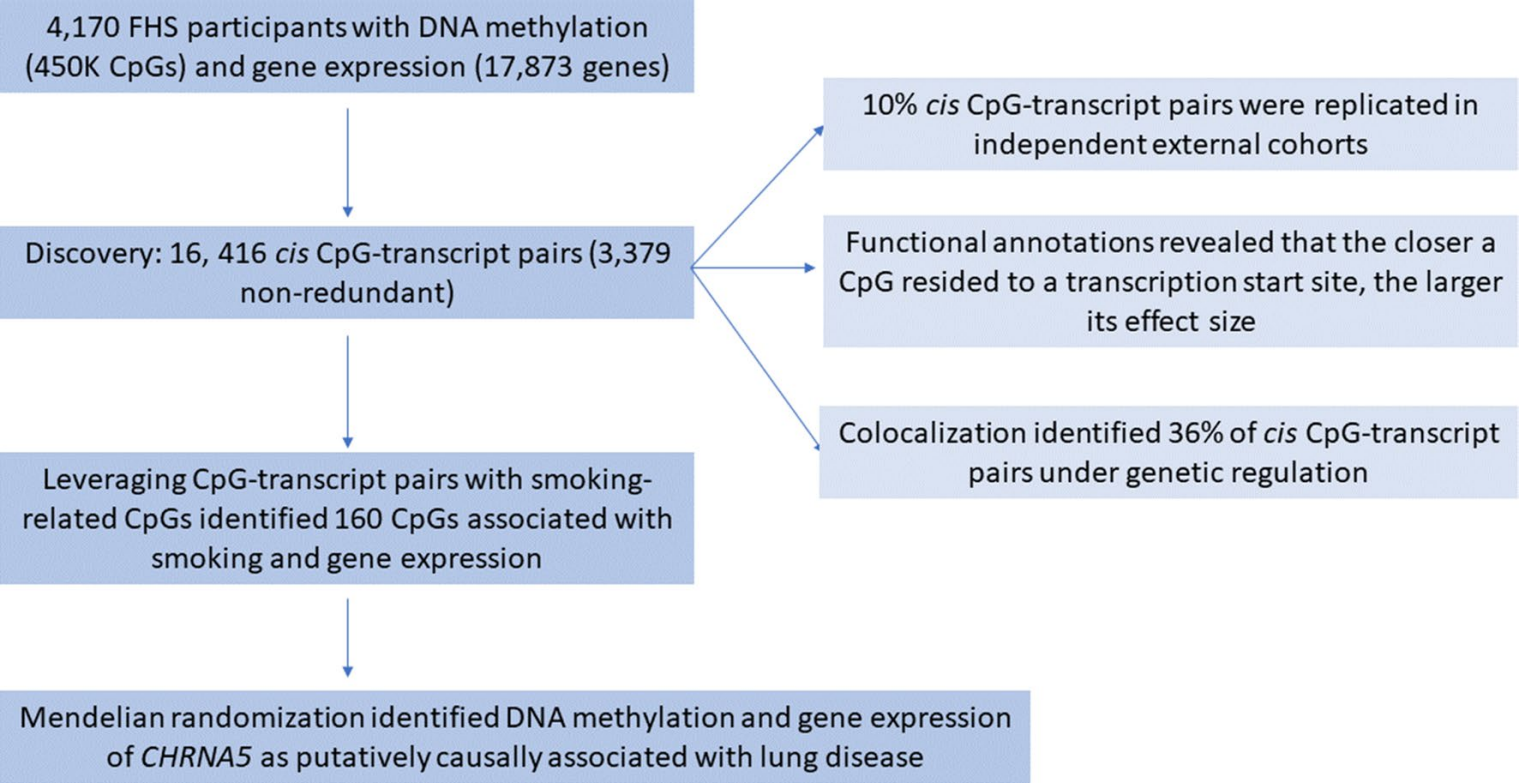
Flowchart of the study design and major findings

To address a knowledge gap regarding how cigarette smoking affects DNA methylation and gene expression and leads to smoking-related disease outcomes, we performed an epigenome-wide association study (EWAS) of whole blood gene expression in ~ 6000 individuals from four cohort studies and identified thousands of CpG-transcript pairs. Our study had the following aims: (i) identify CpGs associated with gene expression (eQTMs), (ii) explore the functional annotations of eQTMs, (iii) conduct colocalization analyses to investigate how genetic influences on DNA methylation contribute to altered gene expression, and (iv) use MR to infer causal effects of CpGs and expressed genes on smoking-related lung diseases (Fig. 1).

## Results

### eQTMs from discovery and replication data sets

To systematically assess the association between DNA methylation and variation in gene expression, we analyzed DNA methylation and genome-wide transcription in whole blood from 4,170 participants in the Framingham Heart Study (FHS). Clinical characteristics of the study sample are summarized in Additional file 2: Table S1. After adjusting for biological and technical covariates (see Methods for details), we identified 16,416 *cis* CpG-transcript pairs (CpG site and the associated transcript [eGene] located within 500 kb) and 198,960 *trans* CpG-transcript pairs (CpG site residing > 500 kb from the eGene) with statistically significant associations. We found that 3% (12,177) of all 401,189 CpG sites tested (see Methods for exclusions of CpGs) were *cis* eQTMs and were associated with expression levels of 15% (2704) of all 17,873 transcripts studied (*P* ≤ 1E−8, Bonferroni correction, Additional file 2: Table S2). Another 6% (24,992) of all 401,189 CpG sites tested were *trans* eQTMs and were associated with 10% (1713) of all transcripts (*P* ≤ 1E−12, Bonferroni correction, Additional file 2: Table S3).

We sought independent external replication of all significant CpG-transcript pairs from three cohorts with whole blood DNA methylation data and Illumina array-based gene expression: KORA (Kooperative Gesundheitsforschung in der Region Augsburg—Cooperative Health Research in the Region of Augsburg, *n* = 783) study, InCHIANTI (Invecchiare in Chianti, *n* = 500) study, and BLSA (Baltimore Longitudinal Study of Aging, *n* = 150). Following meta-analysis of the three replication studies, 10% of the 16,416 *cis* CpG-transcript pairs from the discovery sample replicated (at *P* < 0.05/16,416), with 98% of pairs from discovery showing consistent directions of effect in the meta-analysis of replication cohorts (1672 Affymetrix probes matched up with 1881 Illumina probes, Additional file 2: Table S4). None of the *trans* CpG-transcript pairs discovered in the FHS replicated in the meta-analysis of the other three cohorts after Bonferroni correction (at *P* < 0.05/198,960) and fewer than 6% replicated (pairwise comparisons) between the Illumina platform cohorts. Therefore, we focused this report on *cis* CpG-transcript pairs. To account for the correlation of CpG sites within the same genomic region, we conducted a conditional analysis to identify non-redundant CpGs for a given transcript (see Methods). After adjusting for nearby CpG sites, we identified 3,379 non-redundant *cis* CpG-transcript pairs (at *P* < 1E−8) that were the focus of subsequent analyses (Additional file 2: Table S5). Due to the large platform differences and the limited sample size of the Illumina cohorts, our subsequent analyses were performed using the 3,379 non-redundant *cis* CpG-transcript pairs from FHS discovery. The meta-analyzed results from all four cohorts are provided in Additional file 2: Tables S6.

### Functional annotations of eQTMs

**Fig. 2:**
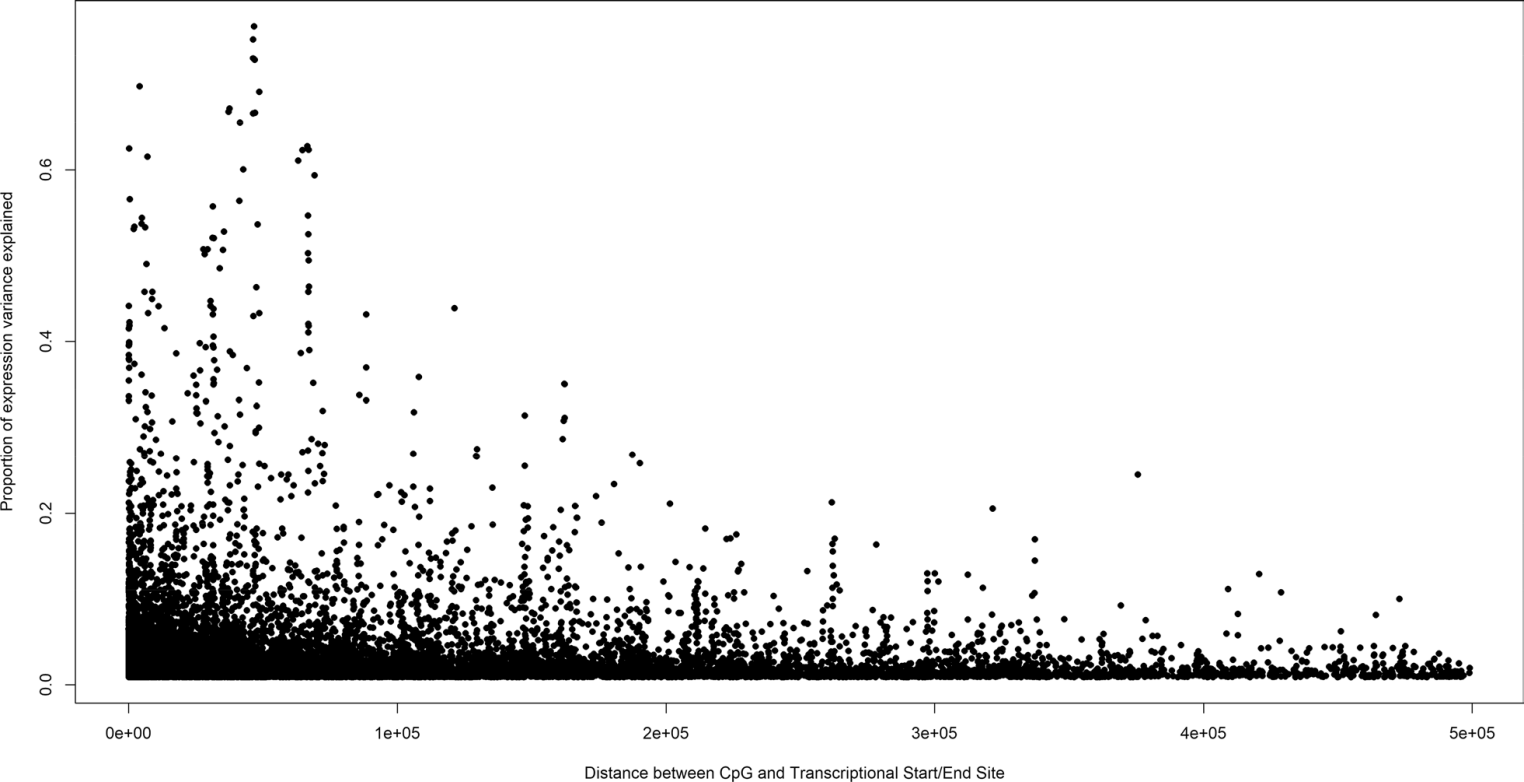
Distance between the CpG site and the transcriptional start or end site vs the proportion of expression variance explained by the CpG. The correlation of distance of the CpG from the transcriptional start site with the proportion of variation in expression explained by the CpG. X-axis is the distance (measured by base pair) between the CpG locus and the transcriptional start/end site of the gene. Y-axis is the proportion of expression variance explained by the CpG (measured by *R*^2^)

The proportion of inter-individual variation in eGene expression explained by *cis* eQTMs ranged from just under 1% to 75%, with a median *R*^2^ of 2%. We found that most CpGs reside in close proximity to their associated transcripts (76% are within 100 kb of the transcription start or end site) and the shorter the distance between a CpG and its paired transcript, the larger the effect size (Pearson correlation *r* = −0.1, *P* = 1.2E−08, Fig. 2).

**Fig. 3:**
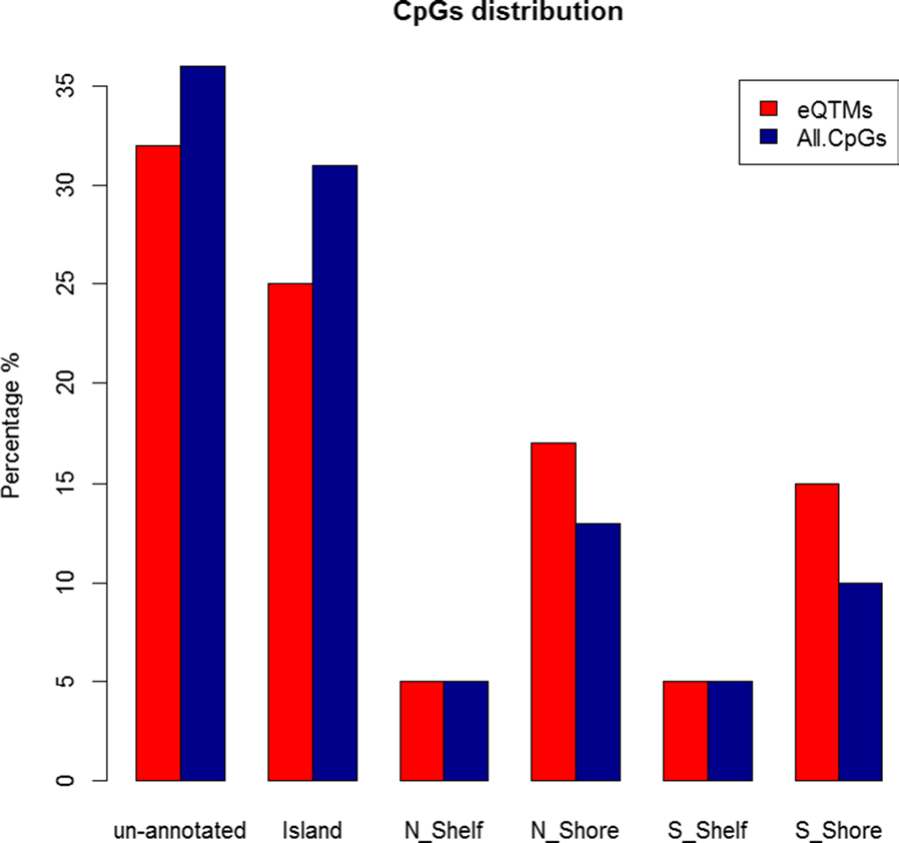
Distribution of *cis* eQTMs in the human genome. Annotation of the *cis* eQTMs from the ENCODE. The percentage of annotation in different functional categories was compared with 450 K CpGs in the array and enrichment was tested using the Chi-square test, which revealed *cis* eQTMs were significantly enriched in CpG island shores, but not in islands (*P* < 1E−4)

**Fig. 4:**
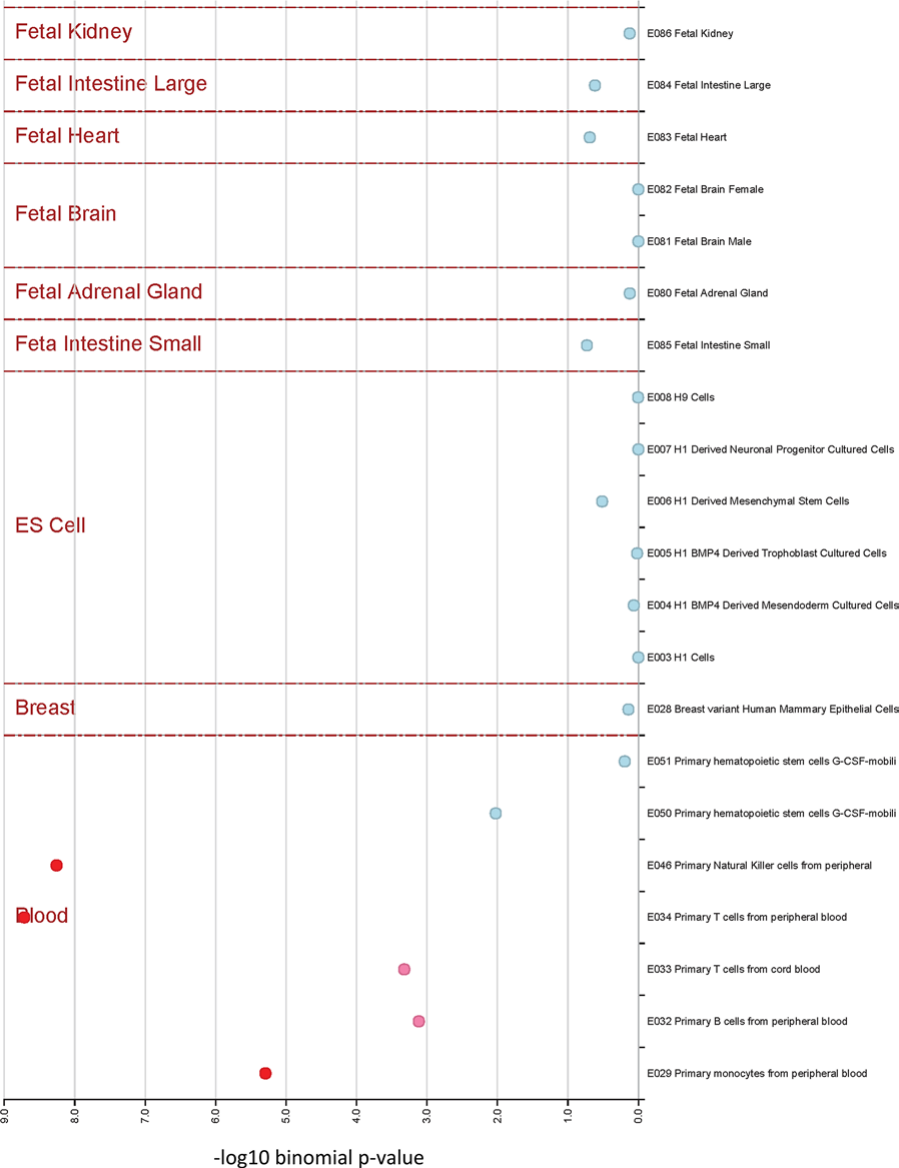
Enrichment of DNase 1 hypersensitive sites (DHSs) in *cis* eQTMs across multiple tissues. Tissue-specific regulatory enrichment of *cis* eQTMs across 21 cell lines using eFORGE

We found that *cis* eQTMs are significantly enriched in CpG island shores (regions within a short distance from the CpG islands, *P* < 1E−4, Chi-square test) but not islands (regions with a high frequency of CpG sites, Fig. 3), and no significant enrichment was found in enhancers. We conducted further annotation using eFORGE (experimentally derived Functional Element Overlap analysis of ReGions from EWAS) [15] to view tissue-specific regulatory components of *cis* eQTMs across 21 cell lines. We found that *cis* eQTMs are significantly enriched in blood cell lines (monocytes, T cells, and natural killer cells, among others), indicating a highly tissue-specific pattern (Fig. 4).

Among the 3,379 unique, non-redundant *cis* CpG-transcript pairs, we identified 2,264 (67%) with negative and 1115 (33%) with positive associations. To explore whether the negatively and positively associated eQTMs reflect different functions in relation to regulation of gene expression, we performed separate Gene Ontology enrichment analyses [16] for each type of association (Additional file 2: Table S7). Although some genes were enriched in common biological processes such as immune response, of note, the 271 genes that were positively associated with DNA methylation were enriched in negative regulation of biological processes (Fisher's exact test *P* = 1.8E−05), whereas the 594 genes that were negatively associated with DNA methylation were enriched in positive regulation of biological processes (Fisher's exact test *P* = 8.1E−10).

### Colocalization of *cis* eQTMs and eQTLs

DNA methylation can change gene expression without altering DNA sequence. Recent findings suggest that a large portion of this epigenetic regulation is also under genetic control [17]. To identify *cis* CpG-transcript pairs regulated by genetic variants, we conducted a Bayesian test of colocalization using the coloc package in R to test whether a CpG site and its corresponding transcript within the same genomic region shared the same sentinel variant [18] (see Methods). Among the 3,379 non-redundant *cis* CpG-transcript pairs from discovery, 2177 shared at least one SNP for both the corresponding *cis* eQTL variants (gene expression was associated with a SNP) [19] and *cis* mQTL variants (DNA methylation was associated with a SNP) in FHS participants at FDR < 0.05. Using all SNPs shared by CpG sites and their corresponding transcripts, we conducted a colocalization test for each pair to determine the probability that the two association signals (mQTL and eQTL) were due to the same *cis* variant (see Methods). For 780 (36%) out of 2,177 *cis* CpG-transcript pairs with shared SNPs, we observed a probability of > 80% that an mQTL variant colocalized with an eQTL variant (Additional file 2: Table S8).

### Cigarette smoking and DNA methylation

Many studies have confirmed that environmental exposures can induce epigenetic changes, i.e., alter DNA methylation. To further investigate the epigenetic mechanisms linking exposures to health outcomes, we explored the DNA methylation signatures of cigarette smoking and linked them to alterations in nearby gene expression using the *cis* CpG-transcript pairs from discovery. We previously reported that 2,622 CpG sites were differentially methylated in current versus never smokers [13]. Intersecting these CpG sites with all 16,416 *cis* CpG-transcript pairs from discovery (see Methods, Additional file 2: Table S2), we identified 160 CpGs that differed between current versus never smokers that also are *cis* eQTMs (Fisher's exact test, *P* = 3.3E−16, Additional file 2: Table S9). To explore whether these smoking-related *cis* eQTM sites are under parallel genetic control along with nearby gene expression, we conducted colocalization analysis of *cis* mQTL variants and *cis* eQTL variants for the corresponding CpG-transcript pairs. Among the 109 *cis* CpG-transcript pairs that shared at least one SNP (i.e., an eQTL variant matched a mQTL variant), we identified colocalization (probability > 80%) for 22 *cis* CpG-transcript pairs. Among the 22 *cis* CpG-transcript pairs with colocalizing genetic signals for CpG mQTLs and transcript eQTLs, DNA methylation levels of 14 CpGs were decreased in current versus never smokers (11 of these CpGs were associated with increased gene expression and three with decreased gene expression) and methylation levels of eight CpGs were increased in current smokers (five of these CpGs were associated with increased gene expression and three with decreased gene expression) (Table 1 and Additional file 2: Table S10).

**Table 1:** Colocalization of smoking related eQTMs with eQTLs

CpG	Genes	Colocalization Locus	eQTM-T (directionality)	Number of SNPs*	Probability of colocalization
cg23813257	IL32	16p13.3	− 9.82	66	1.00
cg02532700	PVALB	22q13.1	− 12.20	67	1.00
cg25174412	C12orf75	12q23.3	5.90	30	1.00
cg07027613	RBP5	12p13.31	7.09	117	1.00
cg12619504	MGAT4B	5q35	− 5.91	140	1.00
cg14656441	NDUFS5	1p34.2-p33	6.86	273	1.00
cg01360605	LOC284757	20q13.33	− 7.25	108	1.00
cg09099830	ITGAL	16p11.2	− 6.33	53	1.00
cg13935577	BTBD11	12q23.3	7.35	235	0.99
cg26105649	NTPCR	1q42.2	− 17.18	484	0.99
cg13834112	ANPEP	15q25-q26	7.81	52	0.99
cg26403843	RNF145	5q33.3	− 6.04	81	0.98
cg26724967	IL32	16p13.3	− 17.33	102	0.98
cg16526047	ISG15	1p36.33	− 7.05	41	0.98
cg16649298	WDR60	7q36.3	6.23	198	0.97
cg13707943	FAM102A	9q34.11	− 7.39	214	0.97
cg04521626	PLD2	17p13.1	6.73	102	0.97
cg16608652	B3GALT2	1q31	− 7.50	2	0.97
cg14018141	CD300A	17q25.1	6.98	27	0.95
cg21913886	TMEM51	1p36.21	− 5.85	293	0.92
cg06478823	ACSM3|ERI2	16p13.11	− 5.79	416	0.92
cg11465630	C21orf33	21q22.3	− 7.89	277	0.88

^*^Number of SNPs associated with both DNA methylation and gene expression in the tested genome locus

**Fig. 5:**
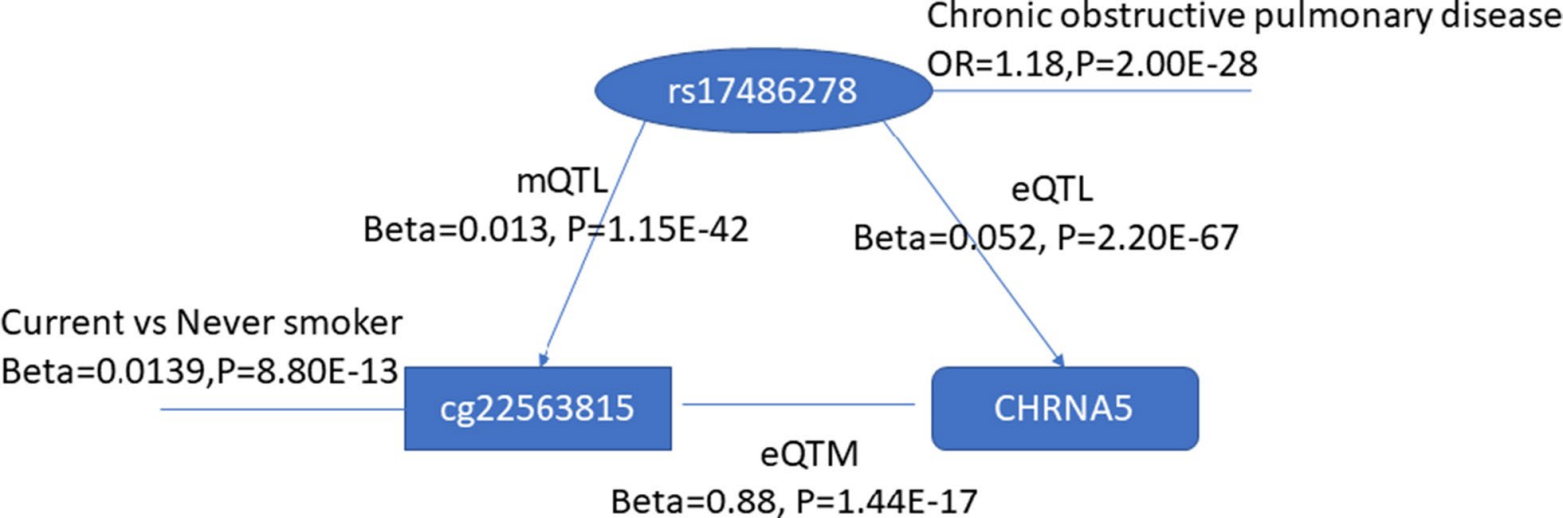
Genetic effects on smoking-related CpG sites and gene expression and the risk of COPD. Relations between genetic variant (rs17486278), DNA methylation (cg22563815) and gene expression (CHRNA5). Cg225638115 was found to be differentially methylated between current smokers and never smokers. This CpG is associated with the expression of *CHRNA5*. The CpG and CHRNA5 both are under genetic control of rs17486278, which has been found to be associated with COPD in previous GWAS

Cigarette smoking is a strong environmental and lifestyle risk factor that is linked to many diseases [20]. To investigate the hypothesis that smoking confers disease risk by altering DNA methylation with resultant effects on expression of key *cis* genes, we intersected the mQTL and eQTL variants associated with *cis* CpG-transcript pairs with SNPs associated with smoking-related diseases from published GWAS [20]. We identified six SNPs that regulate smoking-related CpG sites and that also have been reported to be associated with chronic obstructive pulmonary disease (COPD) and lung cancer—two prominent smoking-related diseases (Table 2). Because these CpG sites also were associated with nearby gene expression (i.e., they are *cis* eQTMs), these results suggest that smoking may promote disease by altering DNA methylation of key CpGs and thereby regulate expression of nearby genes. For example, CpGs in *CHRNA5* have been found to be related to smoking [21]*.* We identified an intronic variant, rs17486278, whose C allele was associated with lower DNA methylation of a smoking-associated CpG in *CHRNA5* (cg22563815), with reduced expression of *CHRNA5*, and with increased risk of COPD based on GWAS [22] (Fig. 5).

**Table 2:** Smoking-related disease GWAS SNPs associated with methylation and gene expression

SNPs	Trait	SNP-associated CpG sites	Beta of SNP-CpG association	SNP-associated expression of genes	Beta of SNP–gene expression association	Beta of CpG–gene expression association
rs8034191	Lung cancer	cg19696491	0.011	CHRNA5	0.052	0.71
rs17486278 (rs11858836 rs8034191)	Chronic obstructive pulmonary disease	cg22563815	0.013		0.05	0.88
rs57221529	Lung disease severity in cystic fibrosis	cg26850624	0.030	AHRR	0.26	1.81
rs1056562	Lung adenocarcinoma	cg03234777	0.0097	AMICA1	0.15	1.51

We identified smoking-related CpGs at three gene loci (within 1 Mb of CpG site) that also harbor GWAS signals for COPD or lung cancer (Table 2). To further explore epigenetic links between cigarette smoking and smoking-related lung diseases, we conducted MR [23] using four CpGs at these three gene loci (Table 2)—cg19696491 and cg22563815 for *CHRNA5*, cg03234777 for *AMICA1,* and cg26850624 for *AHRR*—with the methylation of the CpG as the exposure, *cis* mQTLs of these CpGs as the instrumental variables, and lung cancer or COPD as the outcomes [24]. At *P* < 0.05, we found that lower methylation of two CpG sites at the *CHRNA5* locus was associated with increased risk of lung cancers (adenocarcinoma and squamous cell) and COPD, and lower methylation of a CpG in *AMICA1* was associated with higher risk for lung cancer (adenocarcinoma but not squamous cell cancer; Table 3). We found no causal association between methylation of *AHRR* and lung cancer or COPD. The inferred causal relations between DNA methylation and lung cancer were further validated using mQTLs from lung tissue [25] and GWAS from UK Biobank [26] (Table 3). To explore the effects of gene expression of *CHRNA5*, *AMICA1,* and *AHRR* on lung diseases, we conducted MR using expression of these three genes as the exposure, *cis* eQTLs of these genes from FHS whole blood [19] as the instrumental variables, and lung disease traits as the outcomes. At *P* < 0.05, we found that lower expression of *CHRNA5* was associated with higher risk of lung cancer (Table 4), but not for COPD. The inferred causal relation between gene expression and lung cancer was further tested using *cis* eQTLs in lung tissue from GTEx [27] as the instrumental variable, expression of *CHRNA5* as the exposure, and lung cancer as the outcome. This analysis revealed consistent causal effects of *CHRNA5* on lung cancer.

**Table 3:** Mendelian randomization results of lung cancer as the outcome using DNA methylation as the exposure

Outcome	Exposure	Beta	se	*P* value	Tissue origin
Lung cancer	cg03234777	− 3.81	1.24	2.17E − 03	FHS Whole blood
Lung adenocarcinoma	cg03234777	− 4.96	1.95	1.09E − 02	FHS Whole blood
Squamous cell lung cancer	cg03234777	− 1.32	1.83	4.70E − 01	FHS Whole blood
Lung cancer	cg19696491	− 5.28	0.77	5.05E − 12	FHS Whole blood
Lung adenocarcinoma	cg19696491	− 5.65	1.16	1.18E − 06	FHS Whole blood
Squamous cell lung cancer	cg19696491	− 3.93	1.21	1.11E − 03	FHS Whole blood
Lung cancer	cg22563815	− 4.64	0.67	5.05E − 12	FHS Whole blood
Lung adenocarcinoma	cg22563815	− 4.96	1.02	1.18E − 06	FHS Whole blood
Squamous cell lung cancer	cg22563815	− 3.45	1.06	1.11E − 03	FHS Whole blood
Illnesses of father: lung cancer	cg22563815	− 0.081	0.031	8.28E − 03	FHS Whole blood
Illnesses of father: lung cancer	cg19696491	− 0.092	0.035	8.47E − 03	FHS Whole blood
Cancer code self-reported: lung cancer	cg03234777	− 0.010	0.005	3.43E − 02	FHS Whole blood
Lung adenocarcinoma	cg22563815	− 1.569	0.308	3.60E − 07	Lung tissue
Lung cancer	cg22563815	− 1.456	0.203	7.04E − 13	Lung tissue
Squamous cell lung cancer	cg22563815	− 1.127	0.319	4.04E − 04	Lung tissue
Lung adenocarcinoma	cg19696491	− 2.051	0.416	8.35E − 07	Lung tissue
Lung cancer	cg19696491	− 1.929	0.274	1.82E − 12	Lung tissue
Squamous cell lung cancer	cg19696491	− 1.489	0.429	5.25E − 04	Lung tissue

**Table 4:** Mendelian randomization results of lung cancer using gene expression data as the exposure

Outcome	Exposure	Method	Beta	se	*P* value	Tissue origin
Lung cancer	CHRNA5	Inverse variance weighted	− 1.50	0.23	6.80E − 11	FHS Whole blood
Squamous cell lung cancer	CHRNA5	Inverse variance weighted	− 1.23	0.31	9.05E − 05	FHS Whole blood
Lung adenocarcinoma	CHRNA5	Inverse variance weighted	− 1.58	0.42	1.48E − 04	FHS Whole blood
Lung cancer	CHRNA5	Wald ratio	− 0.22	0.03	9.24E − 13	GTEx lung
Lung adenocarcinoma	CHRNA5	Wald ratio	− 0.23	0.05	5.13E − 07	GTEx lung
Squamous cell lung cancer	CHRNA5	Wald ratio	− 0.17	0.05	3.86E − 04	GTEx lung

**Fig. 6:**
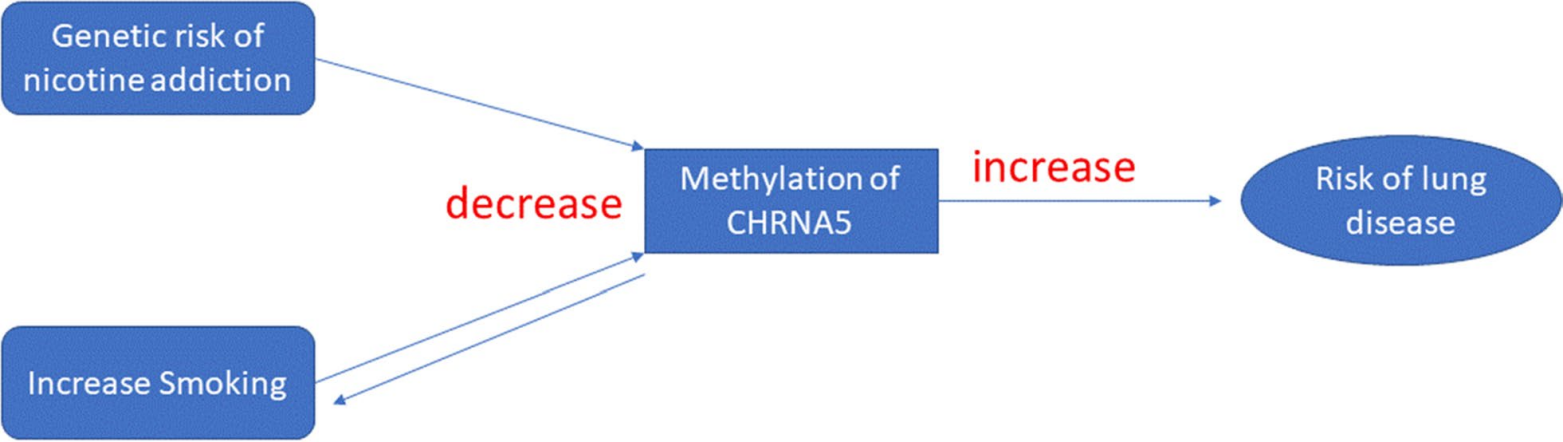
Genetic and environmental effects on methylation of *CHRNA5* in relation to the risk of lung disease. The possible mechanism of methylation of *CHRNA5* affecting the risk of lung disease is through both genetic control and environment influence. The MR test inferred the causal role of methylation of CHRNA5 on lung cancer and COPD. In another bidirectional MR test, the methylation of CHRNA5 and pack-years of smoking played causal roles with each other

Smoking has profound effects on DNA methylation, and the *CHRNA5* locus has been reported to be related to nicotine addiction [28]. To further explore the genetic and environmental effects on *CHRNA5,* we conducted bidirectional MR analyses of methylation of *CHRNA5* in relation to cigarette smoking (see Methods). In the first test (CpG → smoking), we used methylation of CpGs at the *CHRNA5* locus (cg19696491, cg22563815) as the exposures, *cis* mQTLs for these CpGs as the instrumental variables, and pack-years of smoking as the outcome. At *P* < 0.05, we found that reduced methylation of cg19696491/cg22563815 at *CHRNA5* significantly increases smoking exposure in a causal manner. In the second test (smoking → CpG), we used pack-years of smoking as the exposure, pruned GWAS SNPs for smoking from UK Biobank as the instrumental variable, and methylation of *CHRNA5* as the outcome. At *P* < 0.05, we found that greater pack-years of smoking significantly decreases methylation of cg19696491/cg22563815 at *CHRNA5* (Table 5). Bidirectional MR revealed that the association of *CHRNA5* with risk of lung cancer is causally influenced by both genetic and environment effects (Fig. 6).

**Table 5:** Bidirectional Mendelian randomization results of DNA methylation of *CHRNA5* and smoking

Exposure	Outcome	Instrumental variant	Method	beta	se	*P* value
cg19696491	Pack-years of smoking	rs12915652	Wald ratio	− 1.50	0.21	4.94E − 13
cg22563815	Pack-years of smoking	rs12915652	Wald ratio	− 1.32	0.18	4.94E − 13
Pack-years of smoking	cg22563815	9 SNPs	Inverse variance weighted	− 0.077	0.031	0.012
Pack-years of smoking	cg19696491	9 SNPs	Inverse variance weighted	− 0.067	0.026	0.01

## Discussion

We conducted a comprehensive assessment of the association of DNA methylation sites with gene expression and generated a resource of thousands *cis* CpG-transcript pairs that can be used to explore environmentally mediated epigenetic effects on disease. We conducted independent external replication of these findings. We found that the closer a CpG resided to a transcription start site, the larger its effect size is, and that the association of methylation with expression can be positive or negative. Moreover, using colocalization analyses, we found that 36% of *cis* CpG-transcript pairs share the same causal variant (i.e., the mQTL variant for the CpG matches the eQTL variant for the transcript), indicating that in addition to previously described environmental influences on DNA methylation, genetic effects also play an important role in epigenetic regulation. Using these genetic effects as instrumental variables in MR analyses, we identified a putatively causal role of DNA methylation of *CHRNA5* in COPD and lung cancer.

*CHRNA5*, the gene that encodes the acetylcholine receptor subunit alpha 5, has been reported to be associated with tobacco addiction and lung cancer[29]. The possible molecular mechanism has been established by a chrna5 knockout-mouse model, which is analogous to individuals with reduced α5 receptor function[30]. Dramatically increased nicotine consumption was observed in mice with a null mutation in *chrna5*. This effect was reversed in knockout mice by re-expressing α5 subunits in the medial habenula of the mouse brain. These findings suggest that nicotine activates α5-containing nicotinic acetylcholine receptors (nAChRs) to suppress nicotine intake. Our MR results further demonstrate that in humans, genetic variants in *CHRNA5* affect smoking and lung cancer risk through effects on DNA methylation and gene expression of *CHRNA5*.

The low reproducibility rate of *cis* CpG-transcript pairs may be due to two factors. First, the smaller sample size of the replication panel limited the power to replicate. To address this issue, we repeated the discovery-replication experiment in the opposite direction, with discovery from the meta-analysis of KORA, InCHIANTI, and BLSA (4446 *cis* CpG-transcript pairs) and replication in the FHS. This resulted in 57% of discovery *cis* CpG-transcript pairs from the meta-analysis of the three cohorts replicated in the FHS (at *P* < 0.05/4446), which confirmed the first assumption about replication of *cis* CpG-transcript pairs. Second, technical differences in the gene expression platforms (Affymetrix array in FHS versus Illumina array in the other cohorts) may restrict replication. Barnes et al. reported that only 37% of genes had expression levels that were significantly correlated when measuring the same sample using an Affymetrix array versus Illumina gene expression array [31]. To address this, we explored the consistency of results among the cohorts that used an Illumina array for expression profiling and found that 50% (491/987) of the *cis* CpG-transcript pairs from BLSA (the smallest sample size) replicated in the other two cohorts that used the same expression array, but none of them replicated in the FHS (Affymetrix expression array). Similar replication rates were observed among pairwise comparisons among Illumina cohorts, suggesting that platform rather than population differences is critical. On the other hand, the low replication of *trans* CpG-transcript pairs may be due to a lack of power to detect *trans* associations, substantial platform differences (as was the case for *cis* pairs), or because DNA methylation regulates only nearby rather than remote gene expression.

Tobacco exposure is a powerful environmental modifier of DNA methylation [13] and a major risk factor for cancer, cardiovascular disease, COPD, and many other diseases [5–8]. While it is reasonable to hypothesize that environmental factors affect DNA methylation with downstream effects on gene expression that in turn impact disease risk, these relationships are seldom tested formally. In-depth exploration of the inter-relations between genetic variation, DNA methylation, and gene expression is needed to identify mechanisms underlying environmental effects on disease. To that end, we integrated *cis* CpG-transcript pairs with their corresponding mQTLs and eQTLs, which enabled us to identify genetic variants that jointly regulate DNA methylation and gene expression. Finally, we integrated colocalization analysis with large GWAS databases to explore the relations between cigarette smoking and lung diseases. Although many smoking-related CpG sites were not associated with altered gene expression, we identified three genes (*CHRNA5*, *AMICA1,* and *AHRR*) that exhibited interconnected smoking-DNA methylation–gene expression relationships (Table 2). Using MR, we inferred a causal role of lower methylation and lower expression of *CHRNA5* with increased risk for lung cancer (Table 5). For example, carriers of the risk allele for nicotine addiction (rs17486278) have lower methylation of cg19696491/cg22563815 at *CHRNA5*, which increases smoking exposure and the resultant risk of lung cancer. Individuals who do not carry the risk allele, however, may also be at increased risk for lung disease by virtue of smoking-related altered methylation of *CHRNA5*, which in turn increases lung cancer risk. If these findings are tested and validated in the clinical setting, it is possible that they can be used as biomarkers to identify high-risk subgroups (e.g., carriers of the risk allele, those with hypomethylation or reduced expression of CHRNA5) or as therapeutic targets for nicotine addiction treatment.

Causal inference analysis using MR is a powerful tool to distinguish causal from non-causal associations. Our previous cross-sectional study [^17^] reported that current cigarette smoking was associated with increased methylation of cg19696491 (*CHRNA5*), which is opposite to the MR results in the present investigation. When we limited analyses to individuals with fewer than 60 pack-years of smoking, however, we found that pack-years was inversely correlated with methylation of *CHRNA5* (*P* = 0.0006, Additional file 1: Figure S1), which is consistent with our MR results. We further conducted a longitudinal analysis of DNA methylation changes following smoking cessation among smokers in the FHS who quit during follow-up and found that methylation of cg1969649 (*CHRNA5*) was significantly higher (Beta = 0.01, *P* = 0.036) following cessation than when these individuals smoked.

There are several limitations to our study. First, the discovery and replication cohorts used different gene expression platforms, which impaired our ability to replicate results from discovery. Second, DNA methylation and gene expression were profiled in whole blood, which may not reflect tissue-specific effects of DNA methylation on gene expression. Given the tissue-specific nature of eQTMs, our findings should be confirmed in additional disease-relevant tissues and cell types and validated in future studies. Finally, although colocalization has been proposed as a methodology for describing shared genetic influences [32], it relies on a key assumption of no more than two sentinel SNPs at a given locus, which may be inaccurate in some cases.

Our study is among the first investigations of the role of cigarette smoking on DNA methylation and gene expression and how these effects may promote smoking-related diseases. Taken together, our results show that whereas DNA methylation is an important epigenetic mechanism associated with gene expression, genetic variants play important dual roles in the regulation of DNA methylation and gene expression. We demonstrate that genetic variants associated with CpG-transcript pairs (i.e., mQTLs and eQTLs) can be integrated with smoking-related GWAS variants to improve our understanding of the interplay between environmental effects and lung diseases, facilitating the prioritization of candidate genes implicated in the pathogenesis of disease.

## Conclusions

By integrating genetic and epigenetic data, we found that altered DNA methylation and gene expression of *CHRNA5* have putatively causal effects on lung diseases. Using a bidirectional MR approach, we found evidence that DNA methylation and cigarette smoking have mutual effects on *CHRNA5* that in turn influence risk for lung disease. Our findings highlight *CHRNA5* as a potential therapeutic target for lung diseases and also for smoking cessation. The present study illustrated the potential clinical utility of identifying high-risk individuals by virtue of genetic and epigenetic biomarkers; broader application might be achieved in other tumor types in relation to other environmentally mediated disease processes.

## Methods

### Discovery: Framingham Heart Study (FHS)

The FHS is a community-based prospective study, which consists of three generations of participants starting in 1948. The 4170 participants in this study included FHS Offspring cohort (Exam 7; 1998–2001) and Third Generation cohort (Exam 1; 2002–2005) participants. Gene expression: Whole blood was collected in PAXgene™ tubes (PreAnalytiX, Hombrechtikon, Switzerland) and frozen at − 80 °C. RNA was extracted using the whole blood RNA System Kit (Qiagen, Venlo, Netherlands) and mRNA expression profiling was assessed using the Affymetrix Human Exon 1.0 ST GeneChip platform (Affymetrix Inc, Santa Clara, CA), which contains more than 5.5 million probes targeting the expression of 17,873 genes. The Robust Multi-array Average (RMA) package[33] was used to normalize the gene expression values and remove any technical or spurious background variation. Linear regression models were used to adjust for technical covariates (batch, first principal component, and all probeset mean).

DNA methylation: DNA methylation status was assayed using the Infinium HumanMethylation450 BeadChip (Illumina Inc., San Diego, CA). A total of 2,648 samples from FHS offspring cohort were run in two laboratory batches at the Johns Hopkins Center for Inherited Disease Research (laboratory batch #1) and the University of Minnesota Biomedical Genomics Center (laboratory batch #2). A total of 1,522 samples from the FHS Third generation cohort (laboratory batch #3) were run by Illumina (San Diego, CA, USA). QC and normalization were performed using the DASEN methodology implemented in the wateRmelon R package, and the final β values of each CpG for downstream analysis were output as previously described [13]. DNA methylation probes that contained polymorphic SNPs were removed [34]. Because of X-chromosome inactivation, only probes on autosomal chromosomes were analyzed. A total of 401,189 CpGs were retained for further analysis.

### Replication cohorts

KORA: The KORA (Kooperative Gesundheitsforschung in der Region Augsburg—Cooperative Health Research in the Region of Augsburg) study is a series of independent population-based epidemiological surveys and follow-up studies of participants living in the region of Augsburg, Southern Germany. In the present study, we included 707 participants (356 males and 351 females aged 62–81 years) of the KORA F4 study for whom DNA methylation and gene expression data were available. KORA F4 (2006–2008, *N* = 3080) is a follow-up study of the KORA S4 survey (1999/2001, *N* = 4261). The applied standardized examinations have been described in detail elsewhere [35]. The KORA study has been conducted according to the principles expressed in the Declaration of Helsinki. Written informed consent has been given by each participant. The study was reviewed and approved by the local ethics committee (Bayerische Landesärztekammer).

Gene expression: In the KORA F4 study, gene expression was assessed using the Illumina HumanHT-12_v3 expression BeadChip, as described previously [36]. The gene expression data were quantile normalized and log2 transformed. The gene expression data are available for download at ArrayExpress (E-MTAB-1708).

DNA methylation: Genome-wide DNA methylation in KORA F4 was assessed using the Illumina HumanMethylation450 BeadChip as described elsewhere [37]. In brief, bisulfite converted genomic samples were amplified. After enzymatic fragmentation and application of the samples, the arrays were fluorescently stained and scanned using an Illumina HiScan SQ scanner. Data quality was assessed using GenomeStudio (version 2010.3). The methylation data were preprocessed primarily following the CPACOR pipeline[38]. Background correction was performed using the R package minfi, version 1.6.0 [39] and signals with detection *P* values ≥ 0.01 or with less than three functional beads were set to missing. Observations with less than 95% of CpG sites providing reliable signals were excluded. Finally, data were quantile normalized as described by [38], using the R package limma, version 3.16.5 [40]. Beta values representing the percentage of DNA methylation of a cytosine were calculated as the ratio of the methylated signal over the sum of the methylated and unmethylated signals. Following exclusion of cross-reactive probes [41], there were 442,279 CpG sites for investigation. Missing methylation values were imputed using a k-nearest neighbors approach (*k* = 8). Annotations are based on UCSC Genome Browser on Human Feb. 2009 (GRCh37/hg19) Assembly (https://genome.ucsc.edu/).

### InCHIANTI

The InCHIANTI study [42] is a population-based, prospective study of human aging in the Tuscany area of Italy. A total of 1,455 participants were enrolled at baseline (1998–2000), with follow-up waves every 3 years. Extensive interviews, questionnaires, medical examinations, physical tests and blood samples were taken at every wave. Ethical approval was granted by the Instituto Nazionale Riposo e Cura Anziani institutional review board in Italy, and participants gave informed consent to participate.

Gene Expression: Peripheral blood specimens were collected at wave 4 (year 9, 2008–9) from 712 individuals, using the PAXgene technology to preserve levels of mRNA transcripts as they were at the point of collection[43]. RNA was extracted from peripheral blood samples using the PAXgene Blood mRNA kit (Qiagen, Crawley, UK) according to the manufacturer’s instructions. RNA was biotinylated and amplified using the Illumina® TotalPrep™ -96 RNA Amplification Kit and directly hybridized with HumanHT-12_v3 Expression BeadChips that include 48,803 probes. Image data were collected on an Illumina iScan and analyzed using the Illumina and Beadstudio software (Illumina, San Diego, California, USA) as previously described[44]. All microarray experiments and analyses complied with MIAME guidelines.

DNA Methylation: CpG methylation data were generated for a subset of the InCHIANTI participants. Samples taken at baseline (year 0) and during follow-up wave 3 (year 9, the ‘gene expression’ wave) were analyzed using the Illumina Infinium HumanMethylation450 BeadChip. Briefly, genomic DNA was bisulfite converted using Zymo EZ-96 DNA Methylation Kit, followed by CpG analysis using the aforementioned Illumina 450 k array. Quality control of the samples included exclusion based on sex-discrepancy and call-rate thresholds. Normalization of the arrays was performed using the ‘wateRmelon’[45] R package (specifically the DASEN method), which includes quantile normalization between probe types and arrays. Samples having 5% of sites with a detection *P* value > 0.01 were removed. After exclusions, 506 samples having robust data at two waves (9 years apart) were available for analysis. For more detailed methods, see Holly et al. [46].

### BLSA

The Baltimore Longitudinal Study of Aging (BLSA) study is a population-based study aimed to evaluate contributors of healthy aging in the older population residing predominantly in the Baltimore-Washington DC area [47]. Starting in 1958, participants have been examined every one to four years depending on their age. There are ~ 1000 active participants enrolled in the study including 150 who have DNA methylation and gene expression data and were included in this investigation. The BLSA has continuing approval from the Institutional Review Board (IRB) of Medstar Research Institute.

Gene Expression: Gene expression profiling was conducted using the same processes as the InCHIANTI study. In brief, peripheral blood samples were collected for the purposes of gene expression profiling between April 2008 and September 2012. RNA samples were extracted with PAXGene blood mRNA kits (Qiagen), and gene expression was assessed using the Illumina HumanHT-12 v4 expression BeadChip. Gene expression data were log2-transformed (values less than or equal to 0 were imputed as 1) and quantile normalized for analysis.

DNA methylation: DNA methylation was conducted using a process consistent with that of the InCHIANTI study. Briefly, genomic DNA was extracted from whole blood using Gentra Puregene DNA purification system (Qiagen Inc., Germantown, MD). This was followed by bisulfite conversion using EZ DNA methylation kit (Zymo Research Corp., Irvine, CA), and genome-wide methylation was measured using the Illumina Infinium HumanMethylation450 BeadChip (Illumina Inc., San Diego, CA) following the manufacturer’s protocol. Quality control of the samples included exclusion based on sex-discrepancy and call-rate thresholds. Normalization of the arrays was performed using the ‘wateRmelon’[45] R package (specifically the DASEN method), which includes quantile normalization between probe-types and arrays. Samples having 5% of sites with a detection *P* value > 0.01 were removed.

### Statistical analysis

eQTM analysis: First, we computed the residuals of the DNA methylation values using a linear mixed effect model adjusting for the following covariates: age, sex, Houseman’s white blood cell type proportions [48], DNA methylation-specific technical variables (e.g., chip, row, column). Then we computed 25 surrogate variables (SVs) for the residuals and computed the residuals of the residuals. Second, we performed the same clean-up protocol on the gene expression dataset, adjusting for age, sex, Houseman’s white blood cell type proportions [48], and gene expression-specific technical variables (e.g., batch effect, RNA integrity number). We used surrogate variable analysis (SVA) to identify unknown confounders [48]. We chose the number of surrogate variables (SVs) by comparing the internal replication rate of CpG-transcript pairs using FHS data (splitting the full set into discovery and replication samples). We examined replication with 0, 25, and 50 SVs and found that 25 SVs maximized the internal replication rate, thus we used 25 SVs to compute residuals*.* We then applied this protocol to each cohort. Due to differences in laboratory assays, we allowed each cohort to specify their own technical covariates to minimize technical artefacts. *Cis* was defined as a 500-kb window around the transcript unit.

Conditional eQTM analysis: For each transcript, we performed a conditional analysis by adding the CpG site that is most associated (lowest *P* value) with the transcript in the previous analysis as an independent variable. The same linear model of the previous analysis was used with the added conditional CpG term. Beta coefficients, standard errors, *t* values, and *P* values were then collected.

Meta-analysis: Because only results at *P* < 1e−4 were stored in some cohorts, regular meta-analysis approaches could not be used because they require complete availability of beta and standard error values for all cohorts. If only results for which the values are available are ignored, then meta-analysis results may be an underestimation. To remedy this situation, we used a method called MetaNSUE [49] to properly estimate the beta coefficients, standard errors, and *P* values of unstored/missing results and avoid underestimation. The method MetaNSUE does not distinguish random and fixed effects, but it accounts for the between-study heterogeneity and potential covariates by way of its maximum likelihood technique. This method is implemented in the R package MetaNSUE.

Colocalization analysis: For each CpG-transcript pair, the colocalization analysis involved a two-step procedure. Using FHS *cis* mQTL results, we first identified SNPs associated with CpG sites in 1 Mb region (upstream and downstream). Using FHS *cis* eQTL results [19], we then identified SNPs associated with transcripts in 1 Mb region. To estimate the probability that *cis* eQTLs and *cis* mQTLs residing in the same genomic location shared the same causal variant, we conducted a Bayesian test for colocalization of all pairs using all shared SNPs by the coloc package in R [18]. This method requires specifying a prior probability for a SNP being associated with gene expression only (p1), methylation level only (p2), and with both traits (p12). We applied the default *P* values, with p1 and p2 set to 1E−4, assuming that 1 in 10,000 SNPs are causal for either trait, and p12 was set to 1E−5.

Smoking-related CpGs: 2,622 CpG sites that were differentially methylated in current versus never smokers were derived from our previous publication [13]. Current smokers were defined as people who reported smoking at least one cigarette per day within 12 months prior to the blood draw, former smokers were defined as people who previously smoked at least one cigarette per day, but stopped more than 12 months prior to the blood draw, and never smokers were defined as people who never smoked. Pack-years was calculated based on self-reported average number of cigarettes per day smoked divided by 20 multiplied by the number of years of smoking, with zero assigned to never smokers. Because the smoking-related CpG lists did not account for the correlation among CpGs, we leverage the 2,622 smoking-related CpGs with 16,416 cis CpG-transcript pairs from all 450 K CpGs to maximize the overlap.

Mendelian Randomization: MR uses genetic variation as a natural experiment that mimic randomized control trials to infer causal relations between an exposure and an outcome using genetic data from observational studies and GWAS (Additional file 1: Figure S2). MR has three assumptions: (1) that the instrumental variable is robustly associated with the exposure, (2) that the instrumental variable acts independently of confounders, and (3) that the instrumental variable only influences the outcome via its effect on the exposure. Using SNPs significantly associated with DNA methylation or gene expression as genetic instruments for MR satisfies assumptions 1 and 2. Using only cis-mQTLs and cis-eQTLs as instrumental variables satisfies assumption 3. MR was conducted in TwoSampleMR package[23] using DNA methylation or gene expression as exposure, separately. Two-sample (SNP-outcome association is from published GWAS and SNP-exposure association is from FHS mQTLs or eQTLs) MR was used to identify putatively causal CpG sites or genes for lung cancer. SNPs and lung cancer associations were based on the published GWAS. Instrumental variables (IV) for each CpG or gene were composed of independent *cis* mQTLs or *cis* eQTLs pruned by LD at *r*^2^ < 0.001. For CpGs or genes with only one independent SNP after LD pruning, causal effect estimates were determined using the Wald ratio test. When multiple non-redundant SNPs were present, we conducted multi-SNP MR using inverse-variance weighted estimates. Bidirectional MR was first conducted using DNA methylation as exposure and pack-years of smoking as outcome and then vice versa. Summary statistics for SNP-pack years of smoking associations were obtained from UK Biobank GWAS [50]. Pruned SNPs (LD *r*^2^ < 0.001) were used as instrumental variables, and the associations between SNPs and methylation level were calculated in FHS.

## Supplementary Information

**Additional file 1.** Supplemental Figures.

**Additional file 2.** Supplemental Tables.

## Data Availability

The datasets supporting the conclusions of this article are included within the article and its additional files.

## Abbreviations

eQTMs: Expression quantitative trait methylation sites
CTCF: Transcriptional repressor CCCTC-binding factor
MR: Mendelian randomization
EWAS: Epigenome-wide association study
FHS: Framingham Heart Study
eGene: CpG site and the associated transcript
KORA: Kooperative Gesundheitsforschung in der Region Augsburg—Cooperative Health Research in the Region of Augsburg
InCHIANTI: Invecchiare in Chianti
BLSA: Baltimore Longitudinal Study of Aging
eFORGE: Experimentally derived Functional Element Overlap analysis of ReGions from EWAS
mQTL: DNA methylation was associated with a SNP
eQTL: Gene expression was associated with a SNP
COPD: Chronic obstructive pulmonary disease
GTEx: Genotype-Tissue Expression
GWAS: Genome-wide association studies
