# Supplementary material for: Epigenome-wide association study of whole blood gene expression in Framingham Heart Study participants provides molecular insight into the potential role of CHRNA5 in cigarette smoking-related lung diseases
Source: Clin Epigenetics. 2021 Mar 22;13:60. doi: 10.1186/s13148-021-01041-5 (PMC7986283; doi:10.1186/s13148-021-01041-5)
Supplement: Supplementary file 1 — Additional file 1. Supplemental Figures. [file 13148_2021_1041_MOESM1_ESM.pdf]

Fig 1. Association plot of cg01341801 with expression of *HLA-DRB5*

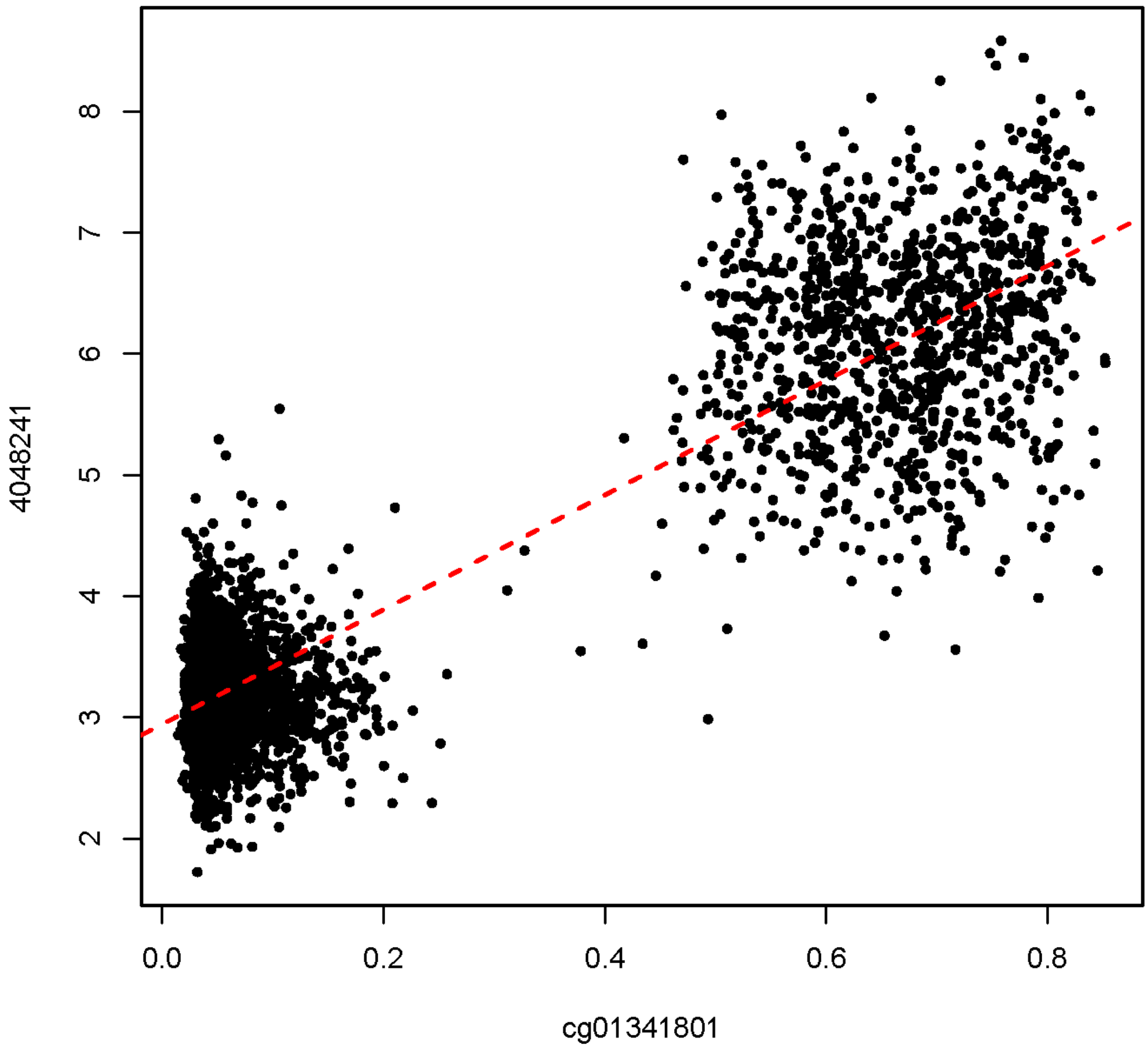

X axis represents the methylation value of cg01341801. Y axis represents the expression value of Affy transcript 4048241 (annotated to *HLA-DRB5*)

Figure H. Association of pack years of smoking with methylation of cg19696491

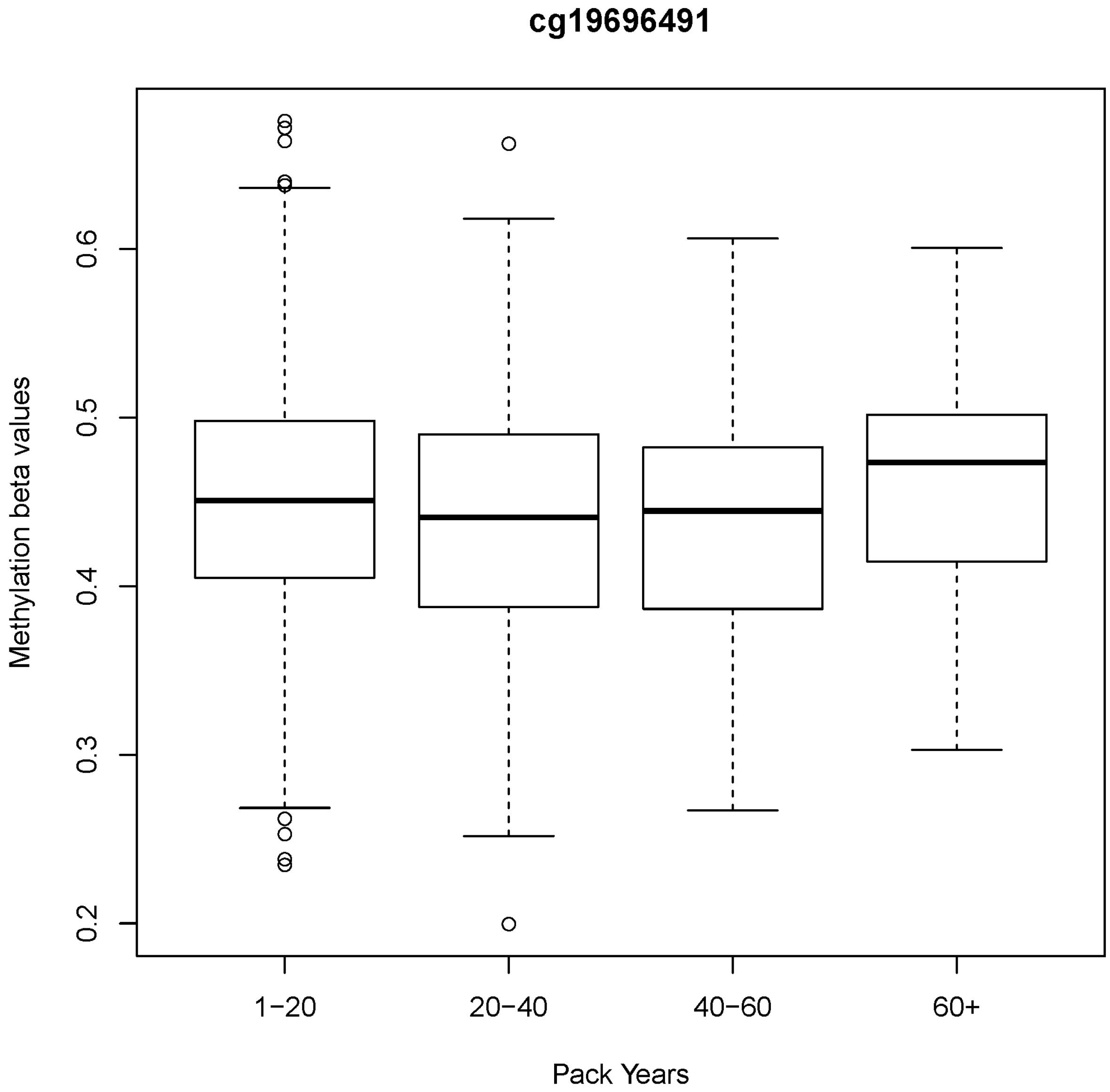

X axis represents the pack years of smoking. Y axis represents the methylation of cg19696491. Pack-years of smoking (<60) was inversely correlated (P=0.0006) with methylation of cg19696491(*CHRNA5*).
